# Postsurgical behaviors in children with and without symptoms of sleep-disordered breathing

**DOI:** 10.1186/2047-0525-3-8

**Published:** 2014-10-14

**Authors:** Alan R Tait, Terri Voepel-Lewis, Louise M O’Brien

**Affiliations:** 1 Department of Anesthesiology, University of Michigan Health System, 1500 E. Medical Center Dr., Ann Arbor, MI 48109, USA; 2 Department of Neurology, University of Michigan Health System, 1500 E. Medical Center Dr., Ann Arbor, MI 48109, USA; 3 Department of Oral/Maxillofacial Surgery, University of Michigan Health System, 1500 E. Medical Center Dr., Ann Arbor, MI 48109, USA; 4 Michael S. Aldridge Sleep Disorders Center, University of Michigan Health System, 1500 E. Medical Center Dr., Ann Arbor, MI 48109, USA

**Keywords:** Pediatric surgery, Postoperative behaviors, Sleep-disordered breathing, Obstructive sleep apnea, Children

## Abstract

**Background:**

Although some children undergo formal preoperative testing for obstructive sleep apnea, it is likely that many children present for surgery with undetected sleep-related disorders. Given that these children may be at increased risk during the perioperative period, this study was designed to compare postoperative behaviors between those with and without symptoms of sleep-disordered breathing (SDB).

**Methods:**

This study represents a secondary analysis of data from a study examining the effect of SDB on perioperative respiratory adverse events in children. Parents of children aged 2–14 years completed the Sleep-Related Breathing Disorder (SRBD) subscale of the Pediatric Sleep Questionnaire prior to surgery. Children were classified as having SDB if they had a positive score (≥0.33) on the SRBD subscale. Seven to ten days following surgery, the SRBD subscale was re-administered to the parents who also completed the Children’s Post Hospitalization Behavior Questionnaire. Children were classified as exhibiting increased problematic behaviors if their postoperative behaviors were considered to be “more/much more” relative to normal.

**Results:**

Three hundred thirty-seven children were included in this study. Children with SDB were significantly more likely to exhibit problematic behaviors following surgery compared with children without SDB. Logistic regression identified adenotonsillectomy (OR 9.89 [3.2–30.9], *P* < 0.01) and posthospital daytime sleepiness (OR 2.8 [1.3–5.9], *P* < 0.01) as risk factors for postoperative problematic behaviors.

**Conclusions:**

Children presenting for surgery with symptoms of SDB have an increased risk for problematic behaviors following surgery. These results are potentially important in questioning whether the observed increase in problematic behaviors is biologically grounded in SDB or simply a response to poor sleep habits/hygiene.

## Background

Sleep disordered breathing (SDB) represents a spectrum of disorders ranging from mild snoring to obstructive sleep apnea syndrome (OSAS) and is thought to afflict approximately 10%–11% of school-aged children [1,2]. Although obesity is the most common risk factor for SDB in adults, the primary cause of SDB in children is a narrowing of the upper airway, most commonly, as a result of adenotonsillar hypertrophy. Clinical manifestations of SDB include periodic episodes of hypopnea, apnea, sleep fragmentation, and arterial oxygen desaturation [3]. Not only can these symptoms affect sleep integrity and contribute to daytime sleepiness but school-aged children with SDB often exhibit neurobehavioral problems including hyperactivity, aggressive behaviors, inattention, and social problems [4-7].

Given the association between adenotonsillar hypertrophy and SDB, many of these children will, at some time, undergo adenotonsillectomy. Indeed, this surgery is considered the treatment of choice for most children with SDB and, other than those who are overweight or obese, has been shown to reverse respiratory morbidity, restore normal sleep patterns, and improve behavioral and neurocognitive function [4,8-10].

Despite the apparent ameliorating effect of adenotonsillectomy on SDB symptoms, children, particularly those with OSAS, undergoing anesthesia and surgery have an increased risk for perioperative adverse events including airway obstruction, oxygen desaturation, and breath-holding [11-16]. Undiagnosed SDB, in particular, may place children at risk for perioperative complications [17]. However, despite the evidence for increased perioperative adverse events among children with SDB, there is a paucity of information regarding how these children respond following anesthesia and surgery. This is important given that recent studies suggest that both typically behaving children and those with attention deficit/hyperactivity disorders (ADHD) may exhibit increased negative behavioral changes that can last up to several weeks following anesthesia and surgery [18-22]. This study was, therefore, designed to compare postoperative behaviors between children with and without symptoms of SDB and to identify risk factors for behaviors deemed problematic. The primary hypothesis tested was that children with a history of SDB would have an increased incidence of postoperative problematic behaviors compared with those without SDB.

## Methods

This study was approved by the University of Michigan’s Institutional Review Board with written informed consent from parents/guardians. The study represents a secondary analysis of data from a primary study evaluating the effects of SDB on perioperative respiratory adverse events in children [16]. The study sample comprised children aged 2 to 14 years of age presenting for an outpatient elective surgical procedure requiring general anesthesia. Children were excluded if they presented with the American Society of Anesthesiologists’ (ASA) physical class 3, 4, or 5, required emergency surgery, were cognitively impairment, or had a history of cardiovascular disease.

Following written informed consent and prior to surgery, parents completed the Sleep-Related Breathing Disorder (SRBD) subscale of the Pediatric Sleep Questionnaire which has been validated for screening in children 2–18 years of age [23]. For the purpose of this study, we utilized the 16-item SRBD subscale which also comprises two four-item subscales for both snoring and daytime sleepiness. The process for scoring has been described elsewhere but, in essence, is based on the number of positive responses out of the number answered. Children with scores of ≥0.33 on the SRBD subscale were considered positive for SDB [23].

### Anesthetic study protocol

Anesthesia management was at the discretion of the anesthesia providers who were blinded to the results of the SRBD questionnaire but may have been aware of some children with previously diagnosed OSA. Demographic data (age, gender, race/ethnicity) were collected prospectively by trained research assistants together with information regarding the type and duration of anesthesia and surgery, co-morbidities (e.g., age- and gender-adjusted body mass index [BMI], asthma, ADHD), and use of perioperative opioids.

### Postoperative follow-up

Parents were telephoned 7–10 days (depending on parent availability) after surgery to evaluate their child’s recovery and behavior since discharge from the hospital. Information was collected by trained research assistants who were blinded to the SDB status of the child. The SRBD subscale used preoperatively was re-administered, and behavioral assessment was measured using the Post Hospitalization Behavior Questionnaire (PHBQ) [24]. The PHBQ measures changes in children’s postoperative behaviors referenced to their normal prehospitalization behaviors and has been validated in children aged 2–13 years [19,20]. This questionnaire consists of 27 items and six categories of anxiety: general anxiety, separation anxiety, sleep anxiety, eating disturbances, aggression against authority, and apathy/withdrawal. Responses are scored on a five-point scale from -2 to +2 reflecting behaviors referenced to normal, i.e., “much less,” “less,” “same,” “more,” and “much more” than before surgery. For the purposes of comparison, behaviors that were scored as either “more” or “much more” than normal were considered to be problematic [19].

### Statistical analysis

Statistical analyses were performed using PASW statistical software (v 18.0, PASW Inc., Chicago, IL). Descriptive data were analyzed using frequency distributions. Comparisons of non-parametric data were performed using Mann-Whitney *U*, chi-square, and Fisher’s exact tests, as appropriate. Parametric data were analyzed using unpaired *t* tests. The positive and negative predictive values of the SRBD tool in predicting problematic behaviors were obtained from 2 × 2 contingency tables. Factors found, by univariate analysis, to be associated with postoperative problematic behaviors as well as those believed to be clinically relevant were entered into a logistic regression model to identify factors predictive of the outcome. Internal consistency of reliability of items in the SRBD and PHBQ tools was measured using the correlation coefficient (Cronbach’s *α*). Cronbach *α* values of >0.7 were considered to represent acceptable internal reliability for the entire SRBD and PHBQ tools and >0.50 for the subscales (due to the smaller number of items) [25].

Data from this study were part of a comprehensive study examining perioperative adverse events in children with and without SDB [16]. As such, the initial sample size for this study was based on a primary outcome of adverse events. The secondary outcome was postoperative behaviors. The sample size used in this study has, however, 90% power to detect what Cohen [26] refers to as a moderate effect size, i.e., 0.6 for postoperative problematic behaviors between children with and without SDB.

## Results

A total of 439 eligible children were approached for inclusion of which 102 either declined or were excluded due to incomplete data. Complete data were thus available for 337 children. Items in the SRBD and PHBQ tools and their respective subscales were tested for internal consistency of reliability. Cronbach *α* values for reliability of the SRBD and PHBQ tools in our sample were 0.74 and 0.84, respectively. Cronbach *α* values for the snoring and sleepiness subscales were 0.82 and 0.60, respectively. Reliability measures of the PHBQ subscales for general anxiety, separation anxiety, eating disturbances, sleep anxiety, apathy/withdrawal, and aggression yielded *α* values of 0.45, 0.71, 0.66, 0.67, 0.55, and 0.78, respectively.

As reported in our previous study [16], just over a quarter of children screened positive for previously undetected SDB. Table 1 describes the demographics of the children with and without a history of SDB. Twelve percent of children in the sample had a prior diagnosis of OSA per parent report. Not surprisingly, results showed that children with SDB were significantly more likely to have undergone adenotonsillectomy compared with children without SDB and were also more likely to be overweight or obese. There were, however, no differences between groups with respect to the number of children with ADHD. Table 2 compares the anesthetic and pain management of the children between groups. Despite the fact that it was not possible to standardize the anesthetic protocols of all the varied surgical procedures, there were no differences between groups with respect to anesthetic and pain management.

**Table 1:** Demographics by sleep-related disordered breathing criteria

	**No SDB**	**SDB**^**a**^
	**(*n*** **= 247)**	**(*n*** **= 90)**
Gender (F)	121 (49.0)	38 (42.0)
Age (years)	6.85 ± 3.4	5.82 ± 3.1*
Race:		
Caucasian	207 (83.8)	69 (76.7)
Black	14 (5.7)	10 (11.1)
Hispanic	8 (3.2)	5 (5.6)
ASA physical status (I/II,%)	52/48	31/69*
Medical history:		
ADHD	3 (1.2)	4 (4.4)
Asthma	30 (12.1)	17 (18.9)
Obesity^b^	28 (11.5)	18 (20.5)*

*SDB* sleep-disordered breathing, *ASA* American Society of Anesthesiologists, *ADHD* attention deficit/hyperactivity disorder.

*P < 0.05 vs no SDB.

^a^Scores of ≥0.33 on the SRBD subscale. Data are mean ± SD or *n* (%).

^b^Based on the child’s BMI corrected for age and gender [27].

**Table 2:** Anesthetic and pain management by sleep-related disordered breathing criteria

	**No SDB**	**SDB**^**a**^
Inpatient/outpatient (%)	18.6/81.4	25.6/74.4
Surgical procedure:		
Airway/non-airway (%)	4.5/95.5	58.9/41.1*
Duration of:		
Anesthesia (mins)	72.6 ± 64.7	58.1 ± 41.8*
Surgery (mins)	52.1 ± 57.2	39.5 ± 34.6*
PACU (mins)	108.4 ± 51.9	139.8 ± 78.6*
Anesthetic agents:		
Sevoflurane	229 (92.7)	88 (97.8)
Isoflurane	219 (88.7)	80 (88.9)
Propofol	104 (42.1)	38 (42.2)
Nitrous oxide	236 (95.5)	87 (96.7)
Anxiolytics:		
Midazolam	130 (52.8)	48 (53.3)
Pain control (morphine equivalents):		
Perioperative (mg/kg/h)	0.14 ± 0.18	0.17 ± 0.14
Hospital inpatient (mg/kg)^b^	0.25 ± 0.38	0.28 ± 0.29

*SDB* sleep-disordered breathing, *PACU* postanesthesia care unit.

*P < 0.05 vs no SDB.

^a^Scores of ≥0.33 on the SRBD subscale. Data are *n* (%) and mean ± SD.

^b^Reported as morphine equivalents mg/kg only (hospital length of stay not recorded).

Responses to items in the SRBD tool showed that 76 (87.4%) and 55 (65.5%) parents of children who screened positive for SDB reported that their child snored “more than half the time” and “always” snores, respectively. In addition, 39 (45.3%) parents reported that their child had “trouble breathing,” and 42 (46.7%) noted that they had observed their child stop breathing during the night. Forty-five (50.6%) parents reported that their child had a problem with daytime sleepiness, and 24 (26.7%) also noted that their child’s teacher had reported that their child was sleepy in school.

Examination of the pre- and post-adenotonsillectomy data showed that snoring and sleepiness decreased significantly following surgery. For example, prior to surgery, 89.1% and 54.7% of children scored ≥0.33 on the snoring and sleepiness subscales, respectively, compared with 33.9% and 37.1% following adenotonsillectomy (*P* < 0.05). Non-airway surgery had no effect on these variables. The positive predictive value of the SRBD tool in predicting postoperative maladaptive behaviors was 0.81, and the corresponding negative predictive value was 0.45. Table 3 compares the postoperative behaviors between children with and without SDB. These data show that children with SDB were more likely to exhibit an increase in postoperative problematic behaviors over normal compared with children without SDB. In particular, children with SDB were more likely to exhibit behaviors consistent with anxiety, eating disturbances, apathy, and aggression. Further analysis also revealed that children with a parent report of existing OSA/SDB had significantly higher total PHBQ scores (indicating more problematic postoperative behaviors) than children who were positive and those who were negative for SDB based on SRBD scores (7.42 ± 8.5 vs 4.42 ± 5.4, vs 2.29 ± 3.92, respectively, *P* < 0.05). Exploratory univariate analysis revealed several factors associated with increased problematic behaviors following surgery (Table 4). For the purpose of this analysis, SDB and its components of snoring and daytime sleepiness were each defined as having a score of ≥0.33 on their respective scales [23]. Interestingly, problematic behaviors were associated not only with posthospital snoring and daytime sleepiness, as might be expected, but also with preoperative snoring and sleepiness. All the associated and clinically relevant factors from this initial exploratory analysis were subsequently forced into a logistic regression and included age, gender, overweight/obesity, adenotonsillectomy, anesthesia and surgery duration, use of perioperative opioids, and pre- and posthospital snoring and sleepiness. Analysis of this model revealed that adenotonsillectomy and posthospital daytime sleepiness were both independent predictors of postoperative problematic behaviors (Table 4). However, given the significant contribution of adenotonsillectomy to the model, we also performed a logistic regression on the data from children who did not have adenotonsillectomy or other airway surgeries. This second model revealed that even among children having non-airway surgery, daytime sleepiness remained an independent predictor of postoperative problematic behaviors (OR [95% CI] = 2.45 [1.28–4.67]). With respect to sleepiness, parents who reported that their child “rarely/sometimes” went to bed at the same time each night were significantly more likely to exhibit postoperative problematic behaviors compared with children who “usually” went to bed at the same time (81.8% vs 59.4%, *P* = 0.015). Similarly, children who “rarely/sometimes” were asleep within 20 min of going to bed were significantly more likely to exhibit problematic behaviors after surgery than children who “usually” were asleep within this time frame (70.3% vs 58.3%, *P* = 0.035).

**Table 3:** Comparison of postoperative behaviors from the Post Hospitalization Behavior Questionnaire

**Behavior**	**No SDB**	**SDB**	**OR (95% CI)**
General anxiety subscale:			
Needs a pacifier	7 (3.0)	2 (2.3)	0.76 (0.18–3.30)
Afraid to leave the house with you	4 (1.7)	5 (5.7)	3.49 (0.99–12.32)
Uninterested in things about them	5 (2.1)	3 (3.4)	1.63 (0.42–6.33)
Bites fingernails	1 (0.4)	1 (1.1)	2.70 (0.28–26.15)
Avoids/afraid of new things	6 (2.5)	7 (7.9)	3.31 (1.13–9.69)*
Difficulty deciding	9 (3.8)	9 (10.2)	2.87 (1.13–7.29)*
Irregular bowel habits	40 (16.9)	25 (28.4)	1.94 (1.10–3.44)*
Sucks fingers/thumbs	4 (1.7)	1 (1.1)	0.67 (0.09–4.52)
Separation anxiety subscale:			
Upset when parent leaves	26 (11.0)	16 (18.2)	1.79 (0.92–3.51)
Upset when doctors or hospitals mentioned	28 (11.9)	15 (17.1)	1.53 (0.78–2.99)
Follows parent around the house	21 (8.9)	18 (20.5)	2.63 (1.34–5.18)*
Seeks attention	33 (13.9)	25 (28.4)	2.44 (1.36–4.39)*
Bad dreams/wakes a lot	27 (11.4)	35 (39.8)	5.11 (2.86–9.15)*
Sleep anxiety subscale:			
Fussy going to bed	9 (3.8)	9 (10.2)	2.87 (1.13–7.29)*
Afraid of the dark	6 (2.5)	6 (6.8)	2.81 (0.93–8.50)
Difficulty going to sleep	19 (8.0)	7 (7.9)	0.99 (0.41–2.38)
Eating disturbances subscale:			
Fussy about eating	16 (6.8)	30 (34.1)	7.11 (3.66–13.83)*
Spends time doing nothing/lying around	43 (18.2)	33 (37.5)	2.69 (1.57–4.63)*
Poor appetite	25 (10.6)	41 (46.6)	7.36 (4.09–13.23)*
Aggression against authority subscale:			
Temper tantrums	22 (9.3)	24 (27.3)	3.65 (1.93–6.89)*
Disobeys	20 (8.47)	21 (23.9)	3.39 (1.74–6.57)*
Apathy/withdrawal subscale:			
Wets the bed	10 (4.2)	7 (7.9)	1.95 (0.74–5.14)
Needs help doing things	30 (12.7)	17 (19.3)	1.64 (0.86–3.14)
Lack of interest in games/toys	8 (3.39)	9 (10.2)	3.25 (1.25–8.44)*
Difficult getting child to talk	7 (2.9)	12 (13.6)	5.17 (2.02–13.19)*
Shy with strangers	9 (3.8)	5 (5.7)	1.52 (0.52–4.46)
Breaks toys/objects	7 (2.9)	5 (5.7)	1.97 (0.64–6.06)

Behaviors reported as “more/much more” than normal. Data are *n* (%).

*SDB* sleep-disordered breathing, *OR* odds ratio, *CI* confidence interval.

*P < 0.05.

**Table 4:** Odds ratios for factors associated with postoperative problematic behaviors

	**OR (95% CI)**	
	**Univariate**	**Multivariate**
Young age (<5 years)	1.26 (0.85–1.87)	
Female gender	0.78 (0.53–1.15)	
Overweight/obese	1.97 (1.03–3.79)*	
T & A surgery	7.30 (3.1–17.31)*	9.89 (3.16–30.97)†
Perioperative opioids	1.27 (0.80–2.00)	
SDB^a^	3.35 (1.86–6.03)*	
Pre surgery snoring^a^	3.18 (1.84–5.50)*	
Posthospital snoring^a^	2.42 (1.25–4.69)*	
Pre surgery daytime sleepiness^a^	2.39 (1.37–4.15)*	
Posthospital daytime sleepiness^a^	2.74 (1.53–4.90)*	2.80 (1.31–5.99)†

Behaviors reported as “more/much more” than normal.

*OR* odds ratio, *CI* confidence interval, *T & A* adenotonsillectomy, *SDB* sleep-disordered breathing.

*P < 0.01, †*P* < 0.01 Wald test.

^a^Based on scores of ≥0.33 on the SDB subscale and the snoring and sleepiness subscales, respectively.

## Discussion

The results of our previous study [16] found that a quarter of the children presenting for anesthesia and surgery had symptoms consistent with SDB that, apparently, were not recognized. This is important given the observations that children who were positive for SDB based on SRBD scores had an increased risk of postoperative problematic behaviors compared with children without sleep-related disorders. This is consistent with other studies of non-surgical pediatric populations showing that children with SDB have a greater prevalence of neurobehavioral problems including somatic complaints, hyperactivity, behavioral problems, anxiety, and aggression [4,5,28,29]. Children without SDB who undergo anesthesia and surgery have also been shown to have an increased risk of problematic behaviors which can last up to several weeks postoperatively [18-22]. Among surgical patients, some studies have identified preoperative anxiety as a predictor of postoperative negative behaviors and have shown that these behaviors may be mitigated by premedication with midazolam [30,31]. Although we did not measure anxiety nor were we able to standardize the anesthetic regimen due to the many different surgical procedures involved, the fact that the anesthesiologists were mostly blinded to SDB status and that the anesthetic and pain management of the two groups including the use of midazolam were similar likely minimized any chance of confounding.

Of particular interest in this study was the observation that daytime sleepiness rather than snoring or other features of SDB was independently predictive of postoperative problematic behaviors among both adenotonsillectomy and non-adenotonsillectomy children and, as such, begs the question of whether these behaviors are biologically grounded as a consequence of SDB or simply a response to poor sleep habits or hygiene. Indeed, problematic behaviors were associated not only with posthospital snoring and daytime sleepiness, as might be expected, but also with preoperative snoring and daytime sleepiness. This suggests that preoperative factors whether biological or otherwise likely contribute to the development of problematic behaviors postoperatively. These results are in concert with others suggesting an association between daytime sleepiness and a range of behavioral and cognitive disorders [6,32]. Recently, O’Brien et al. showed that sleepiness, not snoring, was predictive of conduct problems including aggressive behavior and bullying among a cohort of urban public school children [6]. While daytime sleepiness may reflect an underlying sleep pathology, it has also been associated with unsupervised bedtime curfews, chaotic family dynamics, and the use of television and other electronic devices in the child’s bedroom late at night [6,32]. Indeed, it was interesting to note that children in our study who consistently went to bed at the same time each night had fewer problematic behaviors compared with children with inconsistent bedtimes.

The finding that adenotonsillectomy was predictive of increased postoperative behavioral problems was not wholly unexpected given that this type of surgery is often quite painful and can interfere with normal eating patterns for several days after surgery. In this study, analysis of the PHBQ subscales showed that children who underwent adenotonsillectomy exhibited increased anxiety, eating disturbances, and aggression against authority. Indeed, in a study of otherwise healthy children undergoing elective surgical procedures under anesthesia, Karlin et al. identified pain as an independent predictor of problematic behaviors following surgery [33]. In our study, although postoperative pain scores were not recorded, results showed no differences in the pain management of children with and without SDB.

Despite the fact that children who had undergone adenotonsillectomy in our study were observed to exhibit greater problematic behaviors in the week immediately following surgery, studies suggest that over time, these behaviors do tend to resolve [8,28]. Indeed, research shows that impaired neurocognitive function and behaviors in children with SDB and OSAS are at least partially reversible within 3 months to 1 year following adenotonsillectomy [8,28].

There are several limitations to this study that are recognized. First, this is a non-randomized study and as such may be subject to selection bias. However, since subjects were recruited consecutively as they presented for surgery, they were likely representative of the target surgical population. Another potential limitation is that the categorization of SDB was based on a questionnaire rather than a diagnosis by polysomnography. Certainly, from a pragmatic and cost perspective, one would not expect all children with SDB-related symptoms to undergo polysomnography prior to surgery. However, in instances where polysomnography is neither available nor feasible, the SRBD subscale of the Pediatric Sleep Questionnaire has been shown to be both reliable and valid in identifying SDB in children in clinical research [23]. Another potential limitation was that although daytime sleepiness was assessed using valid measures, this study did not examine all causes of sleepiness and, as such, we were unable to distinguish between organic sleepiness and that resulting from poor sleep habits. Finally, although our choice of time to follow-up was based on a previous study which used the PHBQ to evaluate postoperative behaviors in children with ADHD [22], we recognize that a longer time to follow-up may have provided additional information.

## Conclusions

While the association between SDB and behavior problems in children is well known, there is a paucity of data regarding the effect of SDB on postoperative behaviors in children. Given that many children present for anesthesia and surgery with unrecognized symptoms of SDB, these results will be important in alerting parents to the potential for behavioral problems in the postoperative period. Furthermore, the results of this study are important in that they not only show an association between SDB and postoperative behavioral problems but perhaps, more importantly, show that these behaviors may well be mitigated by daytime sleepiness rather than SDB *per se*. Although examination of the root cause of sleepiness in these children was beyond the scope of this study, an understanding of the link between sleep-disordered breathing, sleepiness, and postoperative problematic behaviors will be important in alerting surgeons, anesthesiologists, and parents to the importance of good sleep hygiene before and after surgery and to teachers as a means to anticipate possible negative behaviors in the classroom.

## Abbreviations

SDB: sleep-disordered breathing; OSAS: obstructive sleep apnea syndrome; SRBD: Sleep-Related Breathing Disorder; PSQ: Pediatric Sleep Questionnaire; PHBQ: Post Hospitalization Behavior Questionnaire; ADHD: attention deficit/hyperactivity disorder; ASA: American Society of Anesthesiologists; OR: odds ratio.

## Competing interests

The authors declare that they have no competing interests.

## Authors’ contributions

ART, TVL, and LMO’B were involved in the planning, execution, analysis, and interpretation of the results and preparation of the manuscript. All authors read and approved the final manuscript.
